# Chlorhexidine bathing and health care-associated infections among adult intensive care patients: a systematic review and meta-analysis

**DOI:** 10.1186/s13054-016-1553-5

**Published:** 2016-11-23

**Authors:** Steven A. Frost, Mari-Cris Alogso, Lauren Metcalfe, Joan M. Lynch, Leanne Hunt, Ritesh Sanghavi, Evan Alexandrou, Kenneth M. Hillman

**Affiliations:** 1 Critical Care Research for Innovation & Evidence Translation (CCRICET) Research Group, School of Nursing and Midwifery, Western Sydney University, Sydney, New South Wales Australia; 2 Simpson Centre for Health Services Research, South Western Sydney Clinical School & Ingham Institute of Applied Medical Research, University of New South Wales, Sydney, New South Wales Australia; 3 Department of Intensive Care, Liverpool Hospital, Sydney, New South Wales Australia; 4 Western Sydney University, Campbelltown Campus, Building 7, Locked Bag 1797, Penrith South, New South Wales DC 1797 Australia

## Abstract

**Background:**

Health care-associated infections (HAI) have been shown to increase length of stay, the cost of care, and rates of hospital deaths (Kaye and Marchaim, J Am Geriatr Soc 62(2):306–11, 2014; Roberts and Scott, Med Care 48(11):1026–35, 2010; Warren and Quadir, Crit Care Med 34(8):2084–9, 2006; Zimlichman and Henderson, JAMA Intern Med 173(22):2039–46, 2013). Importantly, infections acquired during a hospital stay have been shown to be preventable (Loveday and Wilson, J Hosp Infect 86:S1–70, 2014). In particular, due to more invasive procedures, mechanical ventilation, and critical illness, patients cared for in the intensive care unit (ICU) are at greater risk of HAI and associated poor outcomes. This meta-analysis aims to summarise the effectiveness of chlorhexidine (CHG) bathing, in adult intensive care patients, to reduce infection.

**Methods:**

A systematic literature search was undertaken to identify trials assessing the effectiveness of CHG bathing to reduce risk of infection, among adult intensive care patients. Infections included were: bloodstream infections; central line-associated bloodstream infections (CLABSI); catheter-associated urinary tract infections; ventilator-associated pneumonia; methicillin-resistant *Staphylococcus aureus* (MRSA); vancomycin-resistant Enterococcus; and *Clostridium difficile*. Summary estimates were calculated as incidence rate ratios (IRRs) and 95% confidence/credible intervals. Variation in study designs was addressed using hierarchical Bayesian random-effects models.

**Results:**

Seventeen trials were included in our final analysis: seven of the studies were cluster-randomised crossover trials, and the remaining studies were before-and-after trials. CHG bathing was estimated to reduce the risk of CLABSI by 56% (Bayesian random effects IRR = 0.44 (95% credible interval (CrI), 0.26, 0.75)), and MRSA colonisation and bacteraemia in the ICU by 41% and 36%, respectively (IRR = 0.59 (95% CrI, 0.36, 0.94); and IRR = 0.64 (95% CrI, 0.43, 0.91)). The numbers needed to treat for these specific ICU infections ranged from 360 (CLABSI) to 2780 (MRSA bacteraemia).

**Conclusion:**

This meta-analysis of the effectiveness of CHG bathing to reduce infections among adults in the ICU has found evidence for the benefit of daily bathing with CHG to reduce CLABSI and MRSA infections. However, the effectiveness may be dependent on the underlying baseline risk of these events among the given ICU population. Therefore, CHG bathing appears to be of the most clinical benefit when infection rates are high for a given ICU population.

**Electronic supplementary material:**

The online version of this article (doi:10.1186/s13054-016-1553-5) contains supplementary material, which is available to authorized users.

## Background

Health care-associated infections (HAI) have been shown to increase length of hospital stay, the cost of care, and rates of hospital deaths [1–4]. Importantly, infections acquired during a hospital stay have been shown to be preventable [5]. Patients cared for in the ICU are at increased risk of HAI due to the invasive nature of many treatments such as mechanical ventilation, urinary catheterisation, and central venous access. Efforts have been made to reduce hospital-acquired infections among adult intensive care patients, including increased hand hygiene, bundles for insertion of vascular access devices, the screening and isolation of patients colonised with multidrug-resistant organisms, and decontaminating the skin with chlorhexidine (CHG) [6, 7].

The skin of patients is considered a major reservoir for pathogens associated with hospital-acquired infections [8], and has been suggested as a potential target for interventions to reduce bacterial burden and subsequent risk of infection. The use of daily CHG bathing in intensive care patients has been advocated to reduce many of the infections in critically ill patients [9]. However, the effectiveness of CHG bathing to reduce ICU infections has varied considerably among published trials, making the effectiveness of CHG bathing in ICU patients uncertain [10]. This variability has been suggested to be associated with the underlying risk of infection among the ICU patients included in the various trials, with the greatest benefit observed among patients with the highest prevalence of infection at baseline [10]. This meta-analysis was therefore undertaken to summarise the effectiveness of CHG bathing among adult intensive care patients in reducing various infections in the ICU, namely: bloodstream infections (BSI); central line-associated bloodstream infections (CLABSI); catheter-associated urinary tract infections (CAUTI); ventilator-associated pneumonia (VAP); methicillin-resistant *Staphylococcus aureus* (MRSA); vancomycin-resistant Enterococcus (VRE); and *Clostridium difficile* (*C-diff*).

## Methods

This meta-analysis was planned, undertaken, and reported using the Preferred Reporting Items for Systematic Reviews and Meta-analyses (PRISMA) guidelines [11].

### Data sources and search strategy

A systematic literature search was undertaken of medical literature databases including MEDLINE, EMBASE, and Cochrane Library published up until March 2016. Keywords and title searches included a combination of: “Chlorhexidine”, “bath$”, “intensive care”, “prevention”, “infection$”, and “effectiveness”. Hand searching of the references of research papers was also undertaken until no new studies were identified.

### Study selection

#### Inclusion and exclusion criteria

The eligibility for inclusion of research papers into in this meta-analysis was considered independently by two authors (SAF and M-CA). Only trials of the effectiveness to reduce infections in adult ICU patients were included. Review papers, non-adult populations, non-ICUs, and papers that did not report the rates per ICU-days at risk were excluded.

### Data extraction and synthesis

Data extracted from each paper included: first authors’ names and publication year, country of study, duration of study, study site, study design (RCT, before and after) type of ICU setting (surgical, medical, mixed, etc.), infection of interest, and number of events and ICU-days at risk.

### Statistical methods

Individual study and combined estimates of the effectiveness of CHG bathing to prevent infections (incidence rate ratios (IRRs)) are presented as forest plots [12]. Heterogeneity of effectiveness between studies was assessed using an *I* ^2^ statistic and *p* < 0.1 was chosen as evidence of statistical heterogeneity. Initial analysis identified the presence of statistical heterogeneity, and therefore summary estimates for fixed effects and random effects (RE) models are presented [13].

Because of the inclusion of both before-and-after and randomised-cluster crossover trials, summary estimates are also presented using the method suggested by Sutton and Abrams [14]. This approach uses a three-level hierarchical model of the heterogeneity between-study design types, in addition to the heterogeneity between individual studies. Contrasting with traditional approaches, Bayesian methods do not use *p* values to assess statistical significance; in a similar manner to the use of confidence intervals (CIs), credible intervals (CrIs) are used to identify the significance of results. For instance, an estimate of effect as a ratio (i.e. a relative risk) with a 95% CrI that does not include the null (1.0) is considered statistically significant. To assess the potential role of baseline risk for the observed heterogeneity between individual studies, a Bayesian meta-regression approach was also used [15].

In this approach, any regression estimates exhibiting a negative relationship between baseline risk and treatment effect are showing that treatment benefit increased when baseline risk of infection was higher among the given ICU population. Traditional meta-analysis models were developed using the R metafor and meta packages [16]. Bayesian models used non-informative priors with the R2Winbugs package from R, and WinBUGS software [17–19]. Also, because of the inclusion of both before-and-after and randomised studies in our meta-analysis, final interpretation of the effectiveness of CHG bathing was based on Bayesian models. Assessment of publication bias was assessed by inspection of funnel plots and a test of plot asymmetry using a weighted linear regression method [20].

## Results

### Search results

The electronic search resulted in 114 potential papers to be included. Following review of the abstract, or the complete paper when required, 17 before-and-after or randomised-cluster crossover trials were included in our analysis. Reasons why papers were excluded from our final analysis were due to being them being review papers, not among adult ICU patients, and trials that included multiple interventions. Characteristics of the 17 studies are presented in Additional file 1: Table S1. Seven of the studies used a cluster-randomised crossover design, and the remaining studies were of a before-and-after design (the study by Huang et al. [21] reports both before-and-after and randomised-cluster results).

Summary estimates of the effectiveness CHG bathing to reduce infections among adult ICU patients are presented in Table 1, and in the forest plot figures (Additional file 2: Figure S1).

**Table 1:** Fixed effects, random effects, Bayesian random effects, and meta-regression estimates of the effectiveness of chlorhexidine bathing

	Summary estimate IRR (95% CI/CrI)			Meta-regression
Outcome	Fixed effects (I)	Random effects (DL)	Bayesian (RE)	Slope (95% CrI)
BSI	0.79 (0.73, 0.86)^a^	0.79 (0.60, 1.03)	0.78 (0.45, 1.23)	–0.125 (–0.180, –0.071)^a^
CLABSI	0.64 (0.56, 0.74)^a^	0.49 (0.35, 0.68)^a^	0.44 (0.26, 0.75)^a^	–0.050 (–0.123, 0.0231)
VAP	0.83 (0.71, 0.98)^a^	0.85 (0.67, 1.07)	0.82 (0.57, 1.25)	–0.026 (–0.055, 0.002)
CAUTI	1.04 (0.91, 1.18)	0.98 (0.79, 1.21)	0.93 (0.45, 1.66)	–0.008 (–0.048, 0.032)
MRSA-C	0.59 (0.53, 0.65)^a^	0.58 (0.47, 0.71)^a^	0.59 (0.36, 0.94)^a^	–0.010 (–0.0154, 0.005)
MRSA-B	0.66 (0.52, 0.84)^a^	0.66 (0.52, 0.84)^a^	0.64 (0.43, 0.91)^a^	–0.155 (–0.364, 0.054)
VRE-C	0.58 (0.47, 0.73)^a^	0.52 (0.33, 0.82)^a^	0.53 (0.20, 1.55)	–0.027 (–0.065, 0.010)
VRE-B	0.45 (0.25, 0.82)^a^	0.63 (0.19, 2.08)	0.53 (0.15, 2.26)	–1.006 (–1.830, –0.182)^a^
*C-diff*	1.04 (0.91, 1.18)	0.98 (0.48, 1.80)	0.93 (0.48, 1.80)	0.008 (–0.048, 0.032)

^a^Estimates with 95% CrI excluding the null (0 for regression, and 1 for IRR)

*IRR* incidence rate ratio, *I* inverse variance, *DL* DerSimonian-Laird, *RE* random effects, *CI* confidence interval from Bayesian models, *CrI* credible interval from Bayesian models, *BSI* bloodstream infection, *CAUTI* catheter-associated urinary tract infections, C-diff *Clostridium Difficile*, *CLABSI* central line-associated bloodstream infection, *MRSA* methicillin-resistant *Staphylococcus aureus*, *MRSA-C* MRSA colonisation, *MRSA-B* MRSA-associated BSI, *VAP* ventilator-associated pneumonia, *VRE* vancomycin-resistant Enterococcus, *VRE-C* VRE colonisation, *VRE-B* VRE-associated BSI

### Bloodstream infections

Daily bathing with CHG was estimated to reduce BSI in the ICU by approximately 20% (Bayesian RE-IRR = 0.79, 95% CrI 0.60, 1.03). Summary estimates for specific study designs were IRR = 0.86 (95% CI 0.41, 1.83) and IRR = 0.79 (95% CI 0.64, 0.97) for before-and-after and RCT studies respectively. Meta-regression of the relationship between risk among the control group and effectiveness of CHG bathing (Table 1), slope = –0.125 (95% CrI –0.180, –0.071).

### Catheter-related bloodstream infections

Daily bathing with CHG was estimated to reduce CLABSI in the ICU by approximately 56% (Bayesian RE-IRR = 0.44, 95% CrI 0.26, 0.75). Summary estimates for specific study designs were IRR = 0.47 (95% CrI 0.32, 0.70) and IRR = 0.50 (95% CrI 0.31, 0.81) for before-and-after and RCT studies respectively. From meta-regression of the relationship between risk among control group and effectiveness of CHG bathing (Table 1), slope = –0.050 (95% CrI –0.123, 0.023).

### Ventilator-associated pneumonia

Daily bathing with CHG was estimated to reduce VAP in the ICU by approximately 18% (Bayesian RE-IRR = 0.82, 95% CrI 0.57, 1.25). Summary estimates for specific study designs were IRR = 0.77 (95% CrI 0.63, 0.95) and IRR = 1.19 (95% CrI 0.66, 2.13) for before-and-after and RCT studies respectively. From meta-regression of the relationship between risk among control group and effectiveness of CHG bathing (Table 1), slope = –0.026 (95% CrI –0.055, 0.002).

### Catheter-related urinary tract infections

Daily bathing with CHG was estimated to reduce CAUTI in the ICU by approximately 7% (Bayesian RE-IRR = 0.93, 95% CrI 0.45, 1.66). Summary estimates for specific study designs were IRR = 0.98 (95% CrI 0.71, 1.35) and IRR = 0.89 (95% CI 0.57, 1.41) for before-and-after and RCT studies respectively. From meta-regression of the relationship between risk among control group and effectiveness of CHG bathing (Table 2), slope = –0.008 (95% CrI –0.048, 0.032).

**Table 2:** Relative risk, baseline risk, risk with treatment, absolute risk reduction, and number needed to treat

Outcome	Relative effect (95% CrI)	Median (IQR) baseline risk per 1000 days	Risk difference per 1000 days (95% CrI)	Number needed to treat
BSI	0.78 (0.45, 1.03)	5 (4–6)	1.1 (3 fewer to 0.1 more)	910
CLABSI	0.44 (0.26, 0.75)^a^	5 (3–9)	2.8 (4 fewer to 1.2 fewer)^a^	360
VAP	0.82 (0.57, 1.25)	10 (5–16)	1.8 (4 fewer to 3 more)	560
CAUTI	0.93 (0.45, 1.66)	8 (2–14)	0.56 (5 fewer to 5 more)	1565
MRSA-C	0.59 (0.36, 0.94)^a^	4 (3–22)	1.64 (3 fewer to 0.2 fewer)^a^	595
MRSA-B	0.64 (0.43, 0.91)^a^	1 (0.2–2)	0.36 (0.6 fewer to 0.1 fewer)^a^	2780
VRE-C	0.53 (0.20, 1.55)	5 (4–15)	2.35 (4 fewer to 3 more)	425
VRE-B	0.53 (0.15, 2.26)	1 (0.5–2)	0.47 (0.9 fewer to 1.3 more)	2130
*C-diff*	0.93 (0.48, 1.80)	1 (0.5–3)	0.07 (0.5 fewer to 0.8 more)	14,290

^a^Estimates with 95% CrI excluding the null (1 for IRR)

*IRR* incidence rate ratio, *CrI* credible interval from Bayesian models, *BSI* bloodstream infection, *CAUTI* catheter-associated urinary tract infections, C-diff *Clostridium Difficile*, *CLABSI* central line-associated bloodstream infection, *MRSA* methicillin-resistant *Staphylococcus aureus*, *MRSA-C* MRSA colonisation, *MRSA-B* MRSA-associated BSI, *VAP* ventilator-associated pneumonia, *VRE* vancomycin-resistant Enterococcus, *VRE-C* VRE colonisation, *VRE-B* VRE-associated BSI

### Methicillin-resistant *Staphylococcus aureus*

Daily bathing with CHG was estimated to reduce MRSA colonisation and bacteraemia in the ICU by approximately 41% and 36%, respectively (MRSA-C Bayesian RE-IRR = 0.59, 95% CrI 0.36, 0.94; and MRSA-B Bayesian RE-IRR = 0.64, 95% CrI 0.43, 0.91). For MRSA colonisation, summary estimates for specific study designs were IRR = 0.52 (95% CrI 0.39, 0.68) and IRR = 0.68 (95% CrI 0.58, 0.80) for before-and-after and RCT studies respectively. From meta-regression of the relationship between risk among control group and effectiveness of CHG bathing to prevent MRSA colonisation (Table 1), slope = –0.010 (95% CrI –0.005, 0.015). For MRSA bacteraemia, summary estimates for specific study designs were Bayesian RE-IRR = 0.68 (95% CrI 0.48, 0.96) and Bayesian RE-IRR = 0.64 (95% CrI 0.44, 0.94) for before-and-after and RCT studies respectively. From meta-regression of the relationship between risk among control group and effectiveness of CHG bathing to prevent MRSA bacteraemia (Table 1), slope = –0.155 (95% CrI –0.364, 0.054).

### Vancomycin-resistant Enterococcus

Daily bathing with CHG was estimated to reduce both VRE colonisation and bacteraemia in the ICU by approximately 37% (VRE-C Bayesian RE-IRR = 0.53, 95% CrI 0.20, 1.55; and VRE-B Bayesian RE-IRR = 0.53, 95% CrI 0.15, 2.26). From meta-regression of the relationship between risk among control group and effectiveness of CHG bathing to prevent VRE colonisation (Table 1), slope = –0.027 (95% CrI –0.065, 0.010). For VRE bacteraemia, from meta-regression of the relationship between risk among control group and effectiveness of CHG bathing (Table 1), slope = –1.01 (95% CrI –1.830, –0.182).

#### *Clostridium difficile*

Daily bathing with CHG was estimated to reduce *C-diff* infection in the ICU by approximately 7% (Bayesian RE-IRR = 0.93, 95% CrI 0.48, 1.80). Summary estimates for specific study designs were IRR = 0.0.98 (95% CrI 0.71, 1.35) and IRR = 0.89 (95% CrI 0.57, 1.41) for before-and-after and RCT studies respectively. From meta-regression of the relationship between risk among control group and effectiveness of CHG bathing (Table 1), slope = 0.008 (95% CrI –0.048, 0.032).

### Publication bias

Inspection of funnel plots and the results of tests of symmetry of included trials suggested no evidence for publication bias.

### Absolute risk reduction and numbers needed to treat

Relative risk, median baseline risk during control periods, risk with treatment, absolute risk reduction, and number needed to treat (NNT) to prevent a single event are presented in Table 2. Median baseline risk (during control periods) ranged from 1/1000 days to 10/1000 days for the various outcomes of interest. The lowest baseline risk was observed among bacteraemia associated with MRSA or VRE (<1 case/1000 days at risk in the ICU), and the highest baseline risk was observed for VAP (median 10/1000 ventilation-days). The lowest NNT was estimated for CHG bathing to prevent CLABSI and VRE colonisation among ICU patients (NNT = 360 and 425, respectively). The highest estimated NNT was for CHG bathing to prevent MRSA bacteraemia and *C-diff* among ICU patients (NNT = 2780 and 14,290, respectively).

## Discussion

This meta-analysis presents a summary of the estimated benefit of CHG bathing to prevent infection in the ICU. CHG bathing was most effective for the prevention of CLABSI among ICU patients, demonstrating a 56% reduction. However, the magnitude of benefit is affected by the underlying risk of CLABSI among ICU populations. Even among an average risk group of five CLABSI per 1000 central-line-days, 360 patients will need to be bathed with CHG to prevent a single event. If the underlying risk of CLABSI is only 1 per 1000 central-line-days than the NNT increases to 1780. Effectiveness was also shown for reducing MRSA colonisation and MRSA bacteraemia. However, even among average baseline-risk populations, the NNT is approximately 600 and 2800, respectively. Because of varying study designs (before-and-after versus randomised crossover trials), there remains uncertainty in the effectiveness of CHG-B to prevent other infections among adults in the ICU.

Previous reviews of daily CHG bathing to reduce infections in the ICU have been undertaken [22–24], and confirm an observed benefit in CHG bathing to reduce CLABSI and MRSA infection in the ICU. There was considerable variation in the baseline risk of infection in the ICU populations included in the included previous reviews. However, there was no attempt to account for the variability due to study design (the heterogeneity between before-and-after compared with the randomised-cluster trials in some cases differed fourfold).

A potential strength of our meta-analysis is the inclusion of a wide range of infections associated with an ICU stay. In particular, we have attempted to account for the uncertainty of average estimates by using a Bayesian approach to account for variation due to study design. We have also presented the potential clinical effectiveness of CHG bathing for various ICU infections by estimating the absolute rates of infection with treatment and therefore the absolute risk reduction and NNT.

Any systematic review and meta-analysis has a potential weakness of missing unpublished trials, and potential individual trial heterogeneity that is difficult to account for in analysis. It is obvious from the published trials that before-and-after trials tend to overestimate effectiveness, and even variation in the length of a randomised trial may affect the ability to detect underlying benefit. The study by Huang et al. [21] reports both before-and-after results and cluster-randomised results for BSI; the estimate of effect is greater for the before-and-after design, when compared with the cluster grouped outcomes—confirming the potential overestimation of the effectiveness of CHG bathing when compared with historical control periods.

The length of the study intervention and the control period have been proposed to suggest the difference between the results obtained by Climo et al. [25] and Noto et al. [26]: in the latter CHG bathing was found to be ineffective, while the former trial (with 6-month intervention period) found CHG bathing effective in reducing multidrug-resistant infections. An intervention period of only 10-weeks, compared to a 10-week control period, used in the study by Noto et al. [26] has been suggested to be insufficient to determine the true impact of CHG bathing on infection rates among adult ICU patients. However, any potential benefit of an intervention must be assessed in relation to the absolute effectiveness among specific populations of patients, taking into account baseline risk.

Another potential limitation of our meta-analysis is inclusion of the study by Huang et al. [21], in which a screening and isolation group (control) was compared with a group universally decolonised using CHG bathing and twice-daily nasal mupirocin. The removal of this study from our analysis does not change our conclusion for the effect of CHG bathing on CLABSI; however, results for MRSA bacteraemia or colonisation become non-significant. Exclusion of this study assumes a significant added effect of mupirocin, and should be considered when interpreting our final conclusions.

The results of our meta-analysis have some important clinical implications. Each ICU must assess the potential benefit of instituting daily CHG bathing to reduce infections in the ICU such as CLABSI and MRSA. For instance, in an ICU with specific bundles to prevent CLABSI, such as those developed by Pronovost [7, 27], baseline rates of CLABSI may be fewer than 1 per 1000 central-line-days, and the NNT in the order of 1800 patients being bathed daily with CHG to prevent one case of CLABSI. Any benefit from the widespread use of CHG bathing in the ICU should consider the prevalence of central line catheters in a given ICU setting. Also, in terms of other types of infection in the ICU such a *C-diff*, UTIs and VAP, many studies included these as secondary outcomes, and may have lacked adequate sample sizes to assess a lesser effectiveness of CHG bathing.

There are concerns regarding the use of daily bathing of ICU patients with CHG. Although CHG bathing aims to reduce HAI, it may promote the emergence of CHG resistance, and increase Gram-negative organism bacteraemia [10]. However, even modest treatment effects should be considered in the context of the seriousness of some of these specific infections among ICU patients.

## Conclusion

Our meta-analysis of the effectiveness of CHG bathing to reduce infections among adults in the ICU has found evidence for the benefit of daily bathing with CHG to reduce CLABSI and MRSA infections in the ICU. However, the effectiveness was dependent on the underlying risk of these events in the given ICU.

## Additional files

Additional file 1: Table S1. Presenting a summary of study characteristics of the 17 trials on daily CHG bathing of ICU patients. (DOCX 35 kb)
Additional file 2: Figure S1. Showing forest plots presenting the effectiveness of CHG bathing in reducing: BSI (**a**), CLABSI (**b**), VAP (**c**), CAUTI (**d**), MRSA colonisation (**e**), MRSA-associated BSI (**f**), VRE colonisation (**g**), VRE-associated BSI (**h**), and *Clostridium difficile* infections (**i**). *FE* fixed effects, *RE* random effects. (PDF 1056 kb)

## Abbreviations

BSI: Bloodstream infection
CAUTI: Catheter-associated urinary tract infections
*C-Diff*: *Clostridium difficile*
CHG: Chlorhexidine gluconate
CI: Confidence interval
CLABSI: Central line-associated bloodstream infection
CrI: Credible interval
HAI: Health care-associated infection
ICU: Intensive care unit
IRR: Incidence rate ratio
MRSA: Methicillin-resistant *Staphylococcus aureus*
NNT: Number needed to treat
RE: Random effects
VAP: Ventilator-associated pneumonia
VRE: Vancomycin-resistant Enterococcus
