# Supplementary material for: Chlorhexidine bathing and health care-associated infections among adult intensive care patients: a systematic review and meta-analysis
Source: Crit Care. 2016 Nov 23;20:379. doi: 10.1186/s13054-016-1553-5 (PMC5120440; doi:10.1186/s13054-016-1553-5)
Supplement: Additional file 1: Table S1. — Presenting a summary of study characteristics of the 17 trials on daily CHG bathing of ICU patients. (DOCX 35 kb) [file 13054_2016_1553_MOESM1_ESM.docx]

**Additional file 1 Table: S1. Summary of study characteristics of the 17 trials on daily CHG bathing of ICU patients**

| **First author (year)** | **Characteristic** | **Description** |
| --- | --- | --- |
| Bleasdale, 2007 [1] | Duration | 12 months |
|  | Country, ICU setting | USA, medical ICU |
|  | CHG bathing method | Impregnated Cloths |
|  | Outcome(s) | BSI, VAP and c-diff |
|  | Study design | Randomised cross-over |
| Camus, 2005 [2] | Duration | 92 days |
|  | Country, ICU setting | France, medical ICU |
|  | CHG bathing method | Bathing |
|  | Outcome(s) | CLABSI, CAUTI and VAP |
|  | Study design | Cluster randomised |
| Climo, 2009 [3] | Duration | 12 months |
|  | Country, ICU setting | USA, six ICUs, medical, surgical and cardiac |
|  | CHG bathing method | bathing |
|  | Outcome(s) | MRSA-C, MRSA-B, VRE-C and VRE-B |
|  | Study design | Before and after |
| Climo, 2013 [4] | Duration | 12 months |
|  | Country, ICU setting | USA, nine ICUs, medical, surgical, cardiac and bone morrow transplant |
|  | CHG bathing method | Impregnated Cloths |
|  | Outcome(s) | BSI, CLABSI, CAUIT and VAP |
|  | Study design | Randomised cross-over |
| Dicks, 2015 [5] | Duration | 4 years |
|  | Country, ICU setting | USA Seventeen Adult ICUs |
|  | CHG bathing method | Impregnated Cloths |
|  | Outcome(s) | BSI, VAP and c-diff |
|  | Study design | Before and after |
| Dixon, 2010 [6] | Duration | 3 months |
|  | Country, ICU setting | USA, surgical ICU |
|  | CHG bathing method | Impregnated Cloths |
|  | Outcome(s) | CLABSI |
|  | Study design | Before and after |
| Evans, 2010[7] | Duration | 12 months |
|  | Country, ICU setting | USA Trauma ICU |
|  | CHG bathing method | Impregnated Cloths |
|  | Outcome(s) | CLABSI, VAP and MRSA-C |
|  | Study design | Before and after |
| Gould, 2007 [8] | Duration | 4 years |
|  | Country, ICU setting | UK mixed medical-surgical ICU |
|  | CHG bathing method | Bathing |
|  | Outcome(s) | MRSA-c and MRSA-B |
|  | Study design | Before and after |
| Holder, 2009 [9] | Duration | 12 months |
|  | Country, ICU setting | USA Nine specialty ICUs |
|  | CHG bathing method | Impregnated Cloths |
|  | Outcome(s) | CLABSI |
|  | Study design | Before and after |
| Huang, 2013[10] | Duration | 40 months |
|  | Country, ICU setting | USA Seventy-four ICUs in forty-three hospitals |
|  | CHG bathing method | Impregnated Cloths |
|  | Outcome(s) | BSI, MRSA-C and MRSA-B |
|  | Study design | Before-and-after, and Cluster randomised |
| Martinez-Resendez 2014[11] | Duration | 18 months |
|  | Country, ICU setting | USA Seventeen ICUs |
|  | CHG bathing method | Impregnated Cloths |
|  | Outcome(s) | VAP and CAUTI |
|  | Study design | Before and after |
| Montecalvo, 2012 [12] | Duration | 28 months |
|  | Country, ICU setting | USA Medical surgical and Respiratory care unit in four community hospitals.\ |
|  | CHG bathing method | Impregnated Cloths |
|  | Outcome(s) | CLABSI |
|  | Study design | Before and after |
| Munoz-Price, 2009[13] | Duration | 2 years |
|  | Country, ICU setting | USA Long-term care acute hospital |
|  | CHG bathing method | Impregnated Cloths |
|  | Outcome(s) | CLABSI and VAP |
|  | Study design | Before and after |
| Noto, 2015[14] | Duration | 12 months |
|  | Country, ICU setting | USA Five ICUs |
|  | CHG bathing method | Impregnated Cloths |
|  | Outcome(s) | CLABSI, CAUTI, VAP, and C-diff |
|  | Study design | Randomised cross-over |
| Popovich, 2009[15] | Duration | 12 months |
|  | Country, ICU setting | USA single medical ICU |
|  | CHG bathing method | Impregnated Cloths |
|  | Outcome(s) | CLABSI, VAP, MRSA-B and MRSA-B |
|  | Study design | Before and after |
| Popovich, 2010[16] | Duration | 26 months |
|  | Country, ICU setting | USA single surgical ICU |
|  | CHG bathing method | Impregnated Cloths |
|  | Outcome(s) | CLABSI, VAP, MRSA-B and MRSA-B |
|  | Study design | Before and after |
| Vernon, 2006 [17] | Duration | 14 months |
|  | Country, ICU setting | USA a single medical ICU |
|  | CHG bathing method | Impregnated Cloths |
|  | Outcome(s) | VRE-C |
|  | Study design | Before and after, single crossover (two CHG periods and one control period) |
