# Supplementary material for: Chlorhexidine bathing and health care-associated infections among adult intensive care patients: a systematic review and meta-analysis
Source: Crit Care. 2016 Nov 23;20:379. doi: 10.1186/s13054-016-1553-5 (PMC5120440; doi:10.1186/s13054-016-1553-5)
Supplement: Additional file 2: Figure S1. — Showing forest plots presenting the effectiveness of CHG bathing in reducing: BSI (a), CLABSI (b), VAP (c), CAUTI (d), MRSA colonisation (e), MRSA-associated BSI (f), VRE colonisation (g), VRE-associated BSI (h), and Clostridium difficile infections (i). FE fixed effects, RE random effects. (PDF 1056 kb) [file 13054_2016_1553_MOESM2_ESM.pdf]

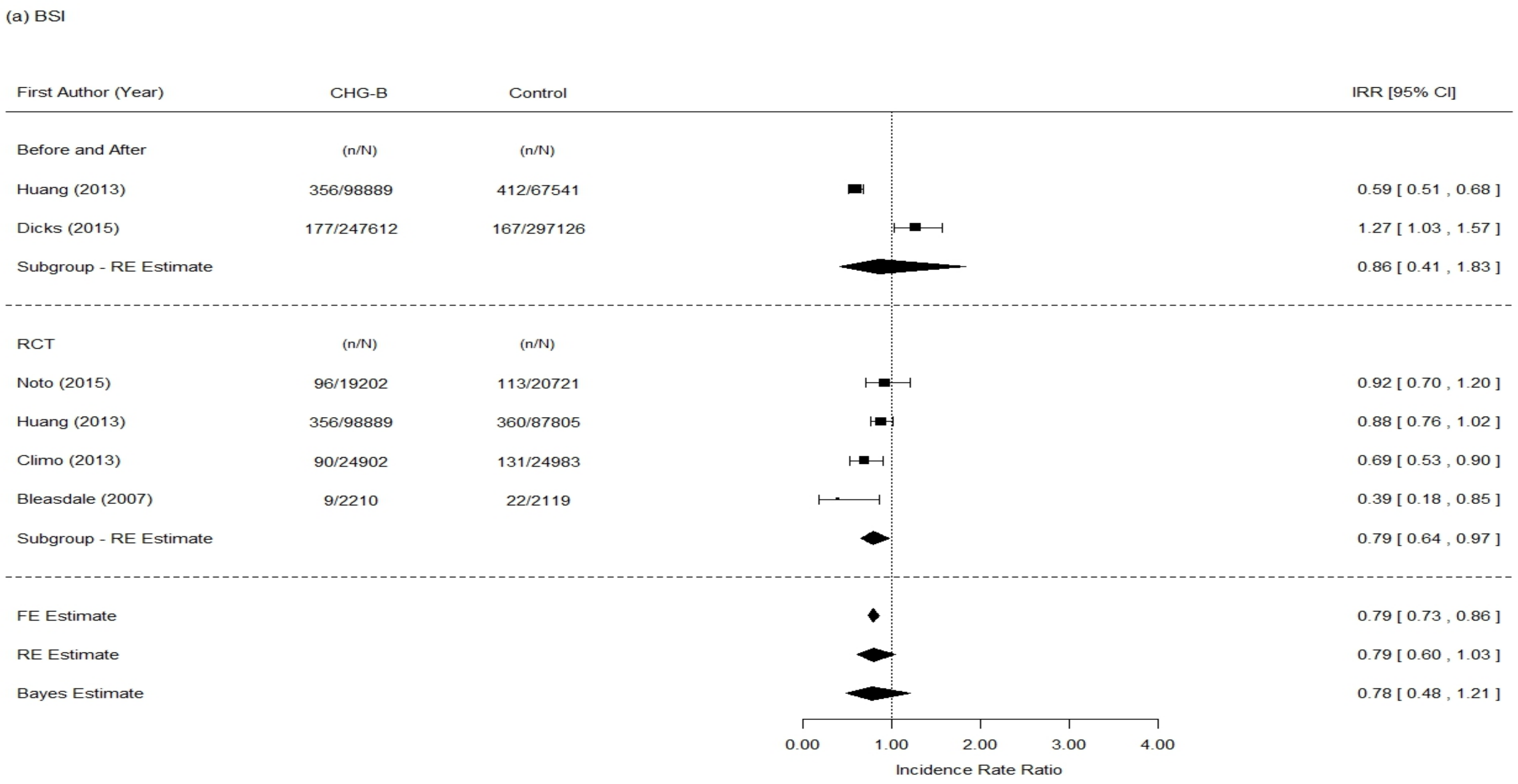

Figure S1. Forest plots presenting the effectiveness of CHG bathing in reducing: BSI (panel A), CLABSI (panel B), VAP (panel C), CAUTI (panel D), MRSA colonisation (panel E), MRSA associated BSI (panel F), VRE colonisation (panel G), VRE associated BSI (panel H), and Clostridium Difficile infections (panel I). FE = fixed effects, RE = random effects.

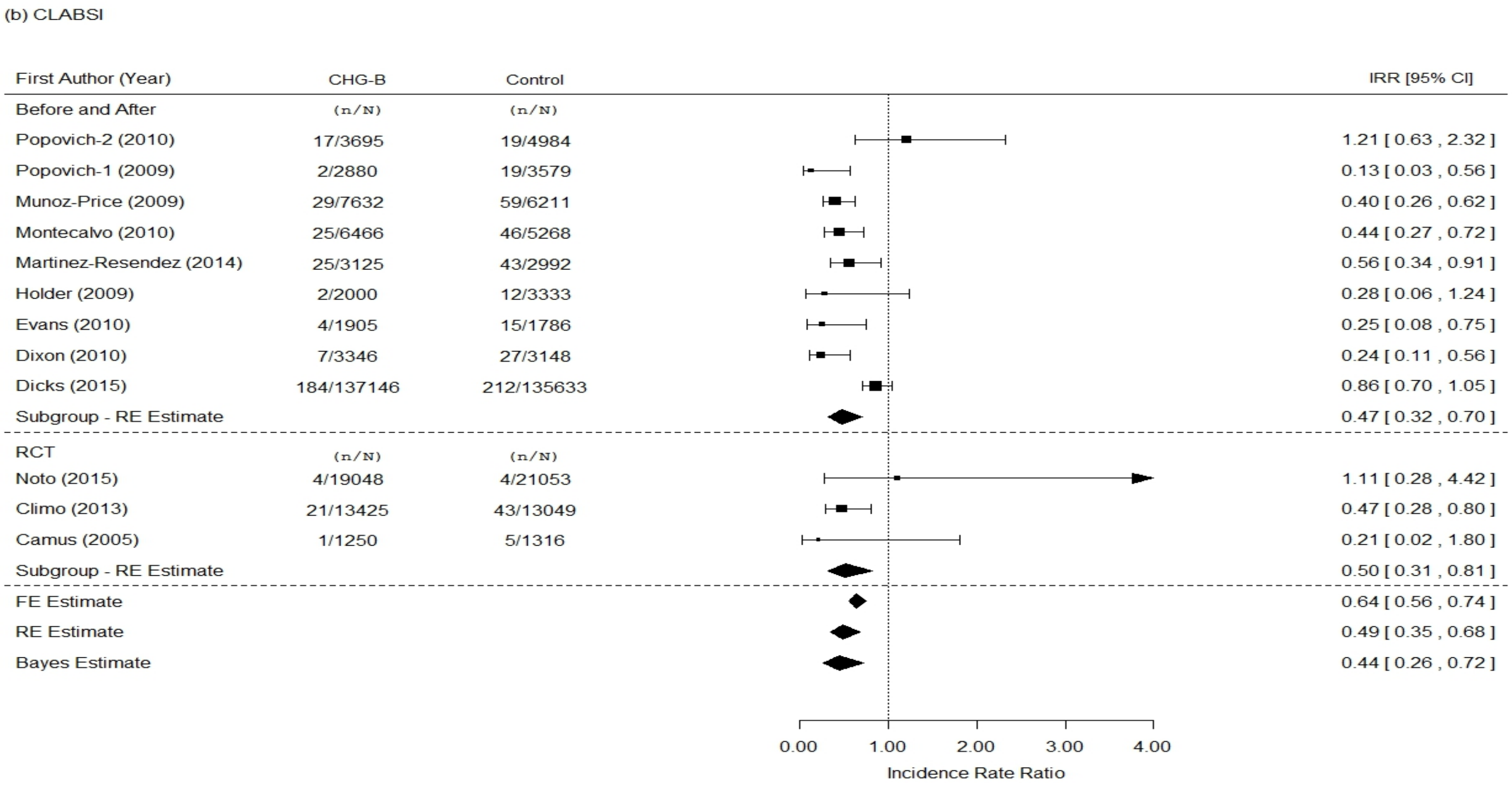

Figure S1. Forest plots presenting the effectiveness of CHG bathing in reducing: BSI (panel A), CLABSI (panel B), VAP (panel C), CAUTI (panel D), MRSA colonisation (panel E), MRSA associated BSI (panel F), VRE colonisation (panel G), VRE associated BSI (panel H), and Clostridium Difficile infections (panel I). FE = fixed effects, RE = random effects.

(c) VAP

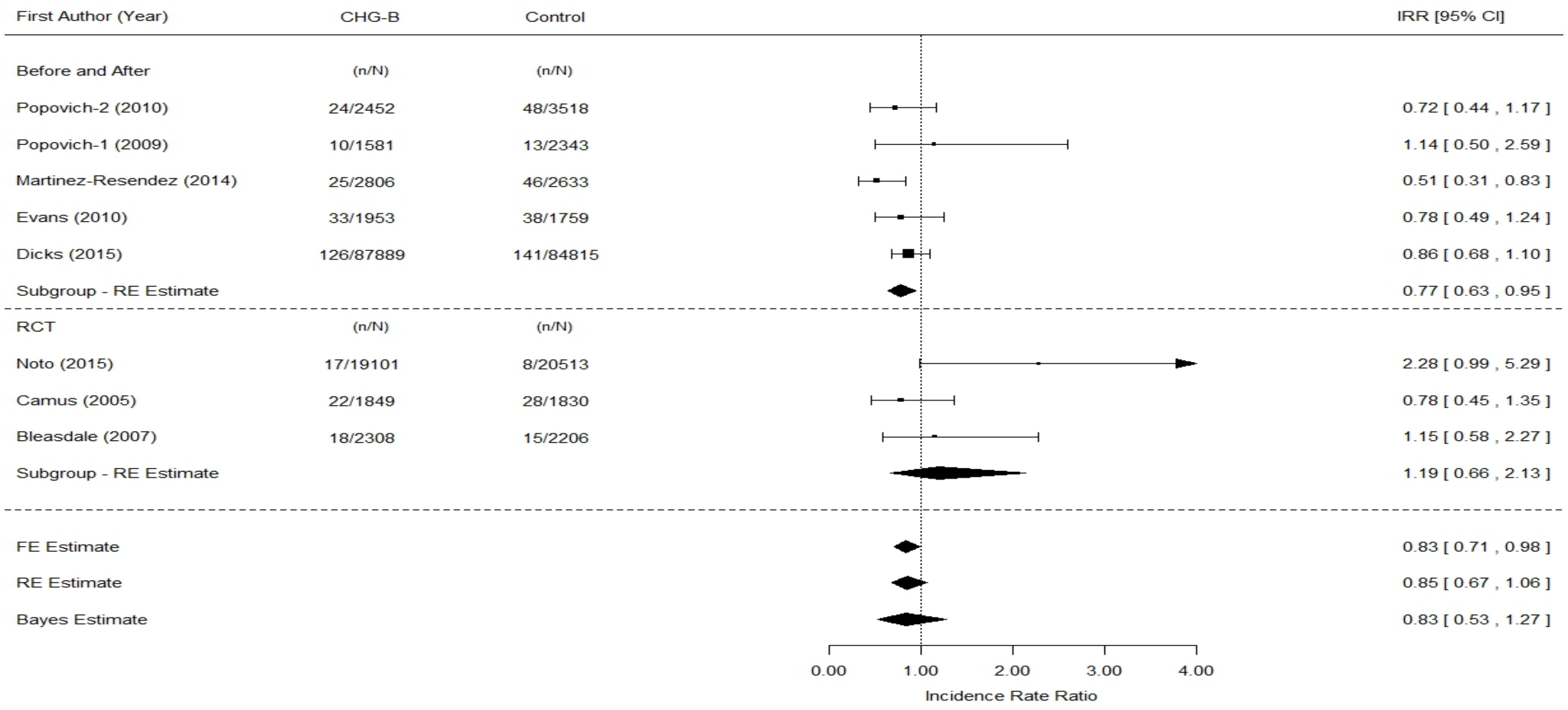

Figure S1. Forest plots presenting the effectiveness of CHG bathing in reducing: BSI (panel A), CLABSI (panel B), VAP (panel C), CAUTI (panel D), MRSA colonisation (panel E), MRSA associated BSI (panel F), VRE colonisation (panel G), VRE associated BSI (panel H), and Clostridium Difficile infections (panel I). FE = fixed effects, RE = random effects.

(d) CAUTI

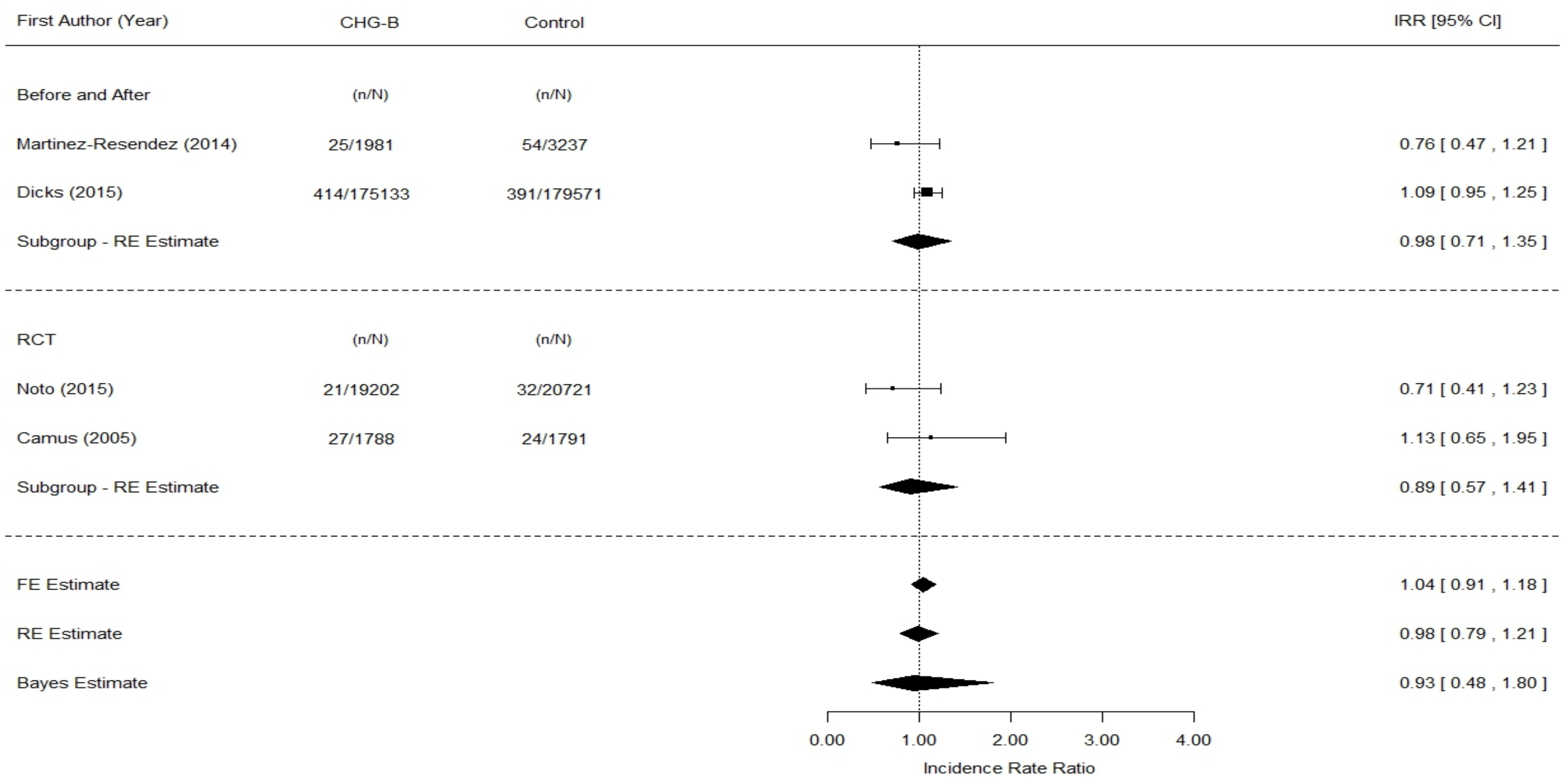

Figure S1. Forest plots presenting the effectiveness of CHG bathing in reducing: BSI (panel A), CLABSI (panel B), VAP (panel C), CAUTI (panel D), MRSA colonisation (panel E), MRSA associated BSI (panel F), VRE colonisation (panel G), VRE associated BSI (panel H), and Clostridium Difficile infections (panel I). FE = fixed effects, RE = random effects.

(e) MRSA-C

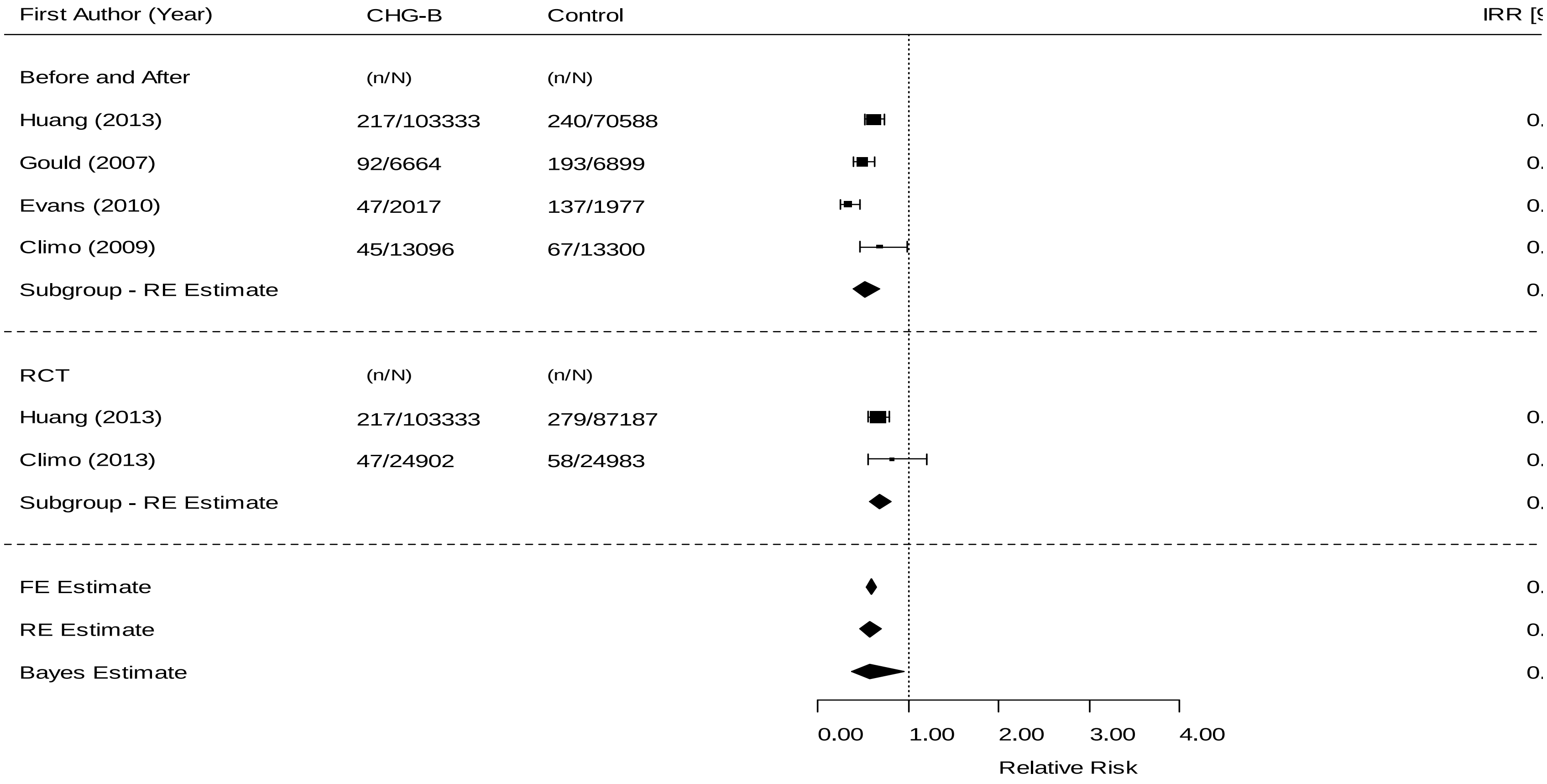

Figure S1. Forest plots presenting the effectiveness of CHG bathing in reducing: BSI (panel A), CLABSI (panel B), VAP (panel C), CAUTI (panel D), MRSA colonisation (panel E), MRSA associated BSI (panel F), VRE colonisation (panel G), VRE associated BSI (panel H), and Clostridium Difficile infections (panel I). FE = fixed effects, RE = random effects.

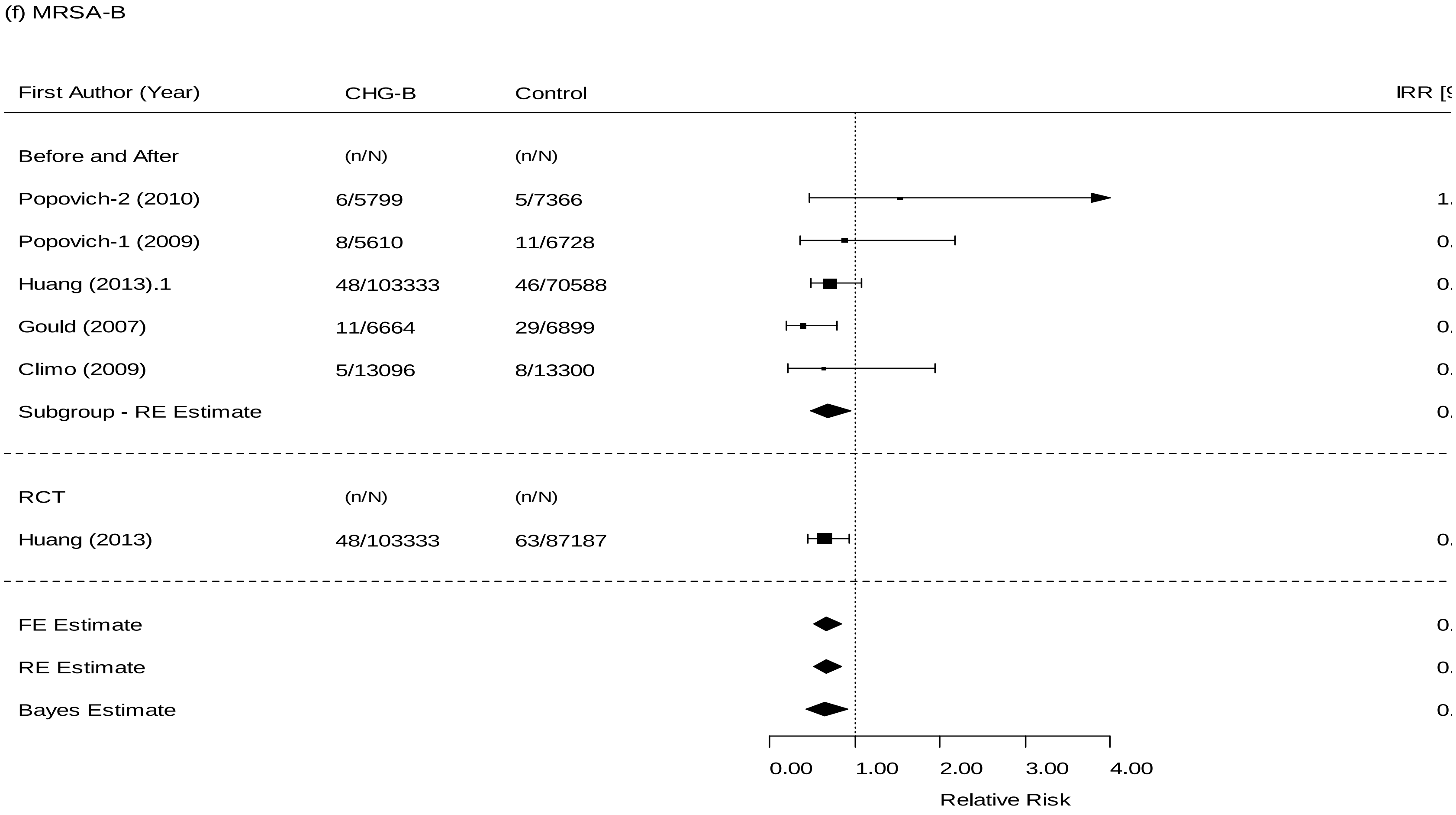

Figure S1. Forest plots presenting the effectiveness of CHG bathing in reducing: BSI (panel A), CLABSI (panel B), VAP (panel C), CAUTI (panel D), MRSA colonisation (panel E), MRSA associated BSI (panel F), VRE colonisation (panel G), VRE associated BSI (panel H), and Clostridium Difficile infections (panel I). FE = fixed effects, RE = random effects.

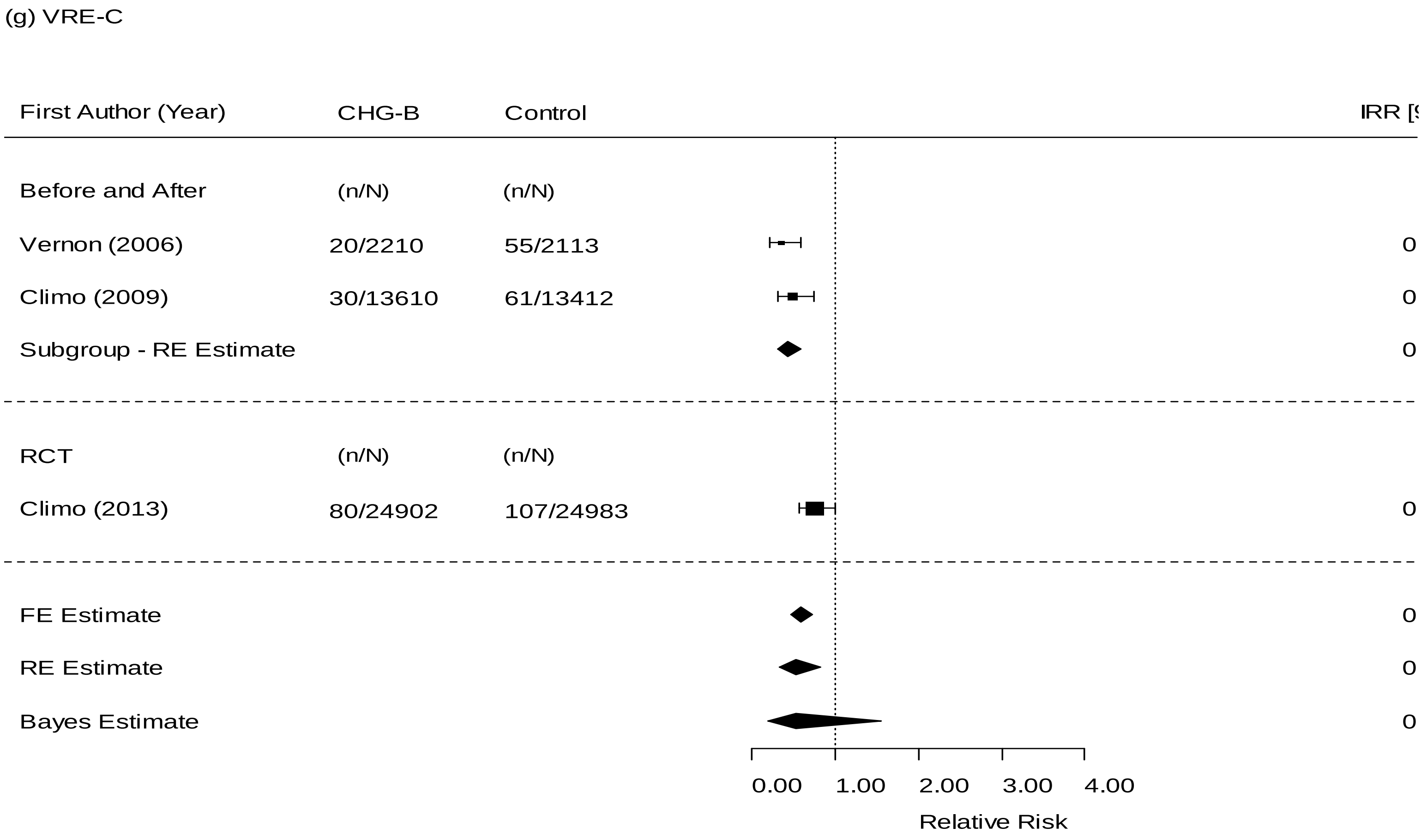

Figure S1. Forest plots presenting the effectiveness of CHG bathing in reducing: BSI (panel A), CLABSI (panel B), VAP (panel C), CAUTI (panel D), MRSA colonisation (panel E), MRSA associated BSI (panel F), VRE colonisation (panel G), VRE associated BSI (panel H), and Clostridium Difficile infections (panel I). FE = fixed effects, RE = random effects.

(h) VRE-B

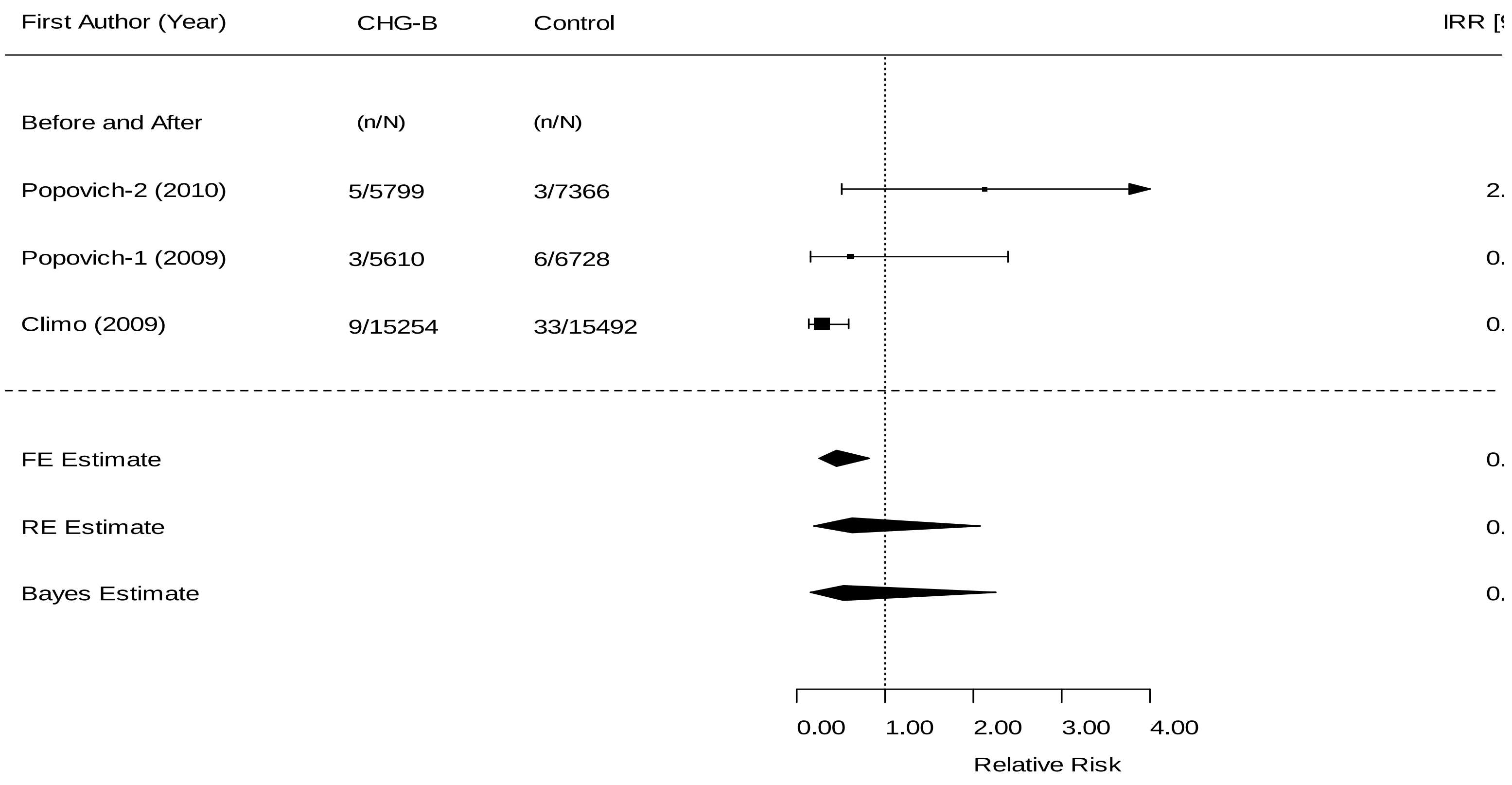

Figure S1. Forest plots presenting the effectiveness of CHG bathing in reducing: BSI (panel A), CLABSI (panel B), VAP (panel C), CAUTI (panel D), MRSA colonisation (panel E), MRSA associated BSI (panel F), VRE colonisation (panel G), VRE associated BSI (panel H), and Clostridium Difficile infections (panel I). FE = fixed effects, RE = random effects.

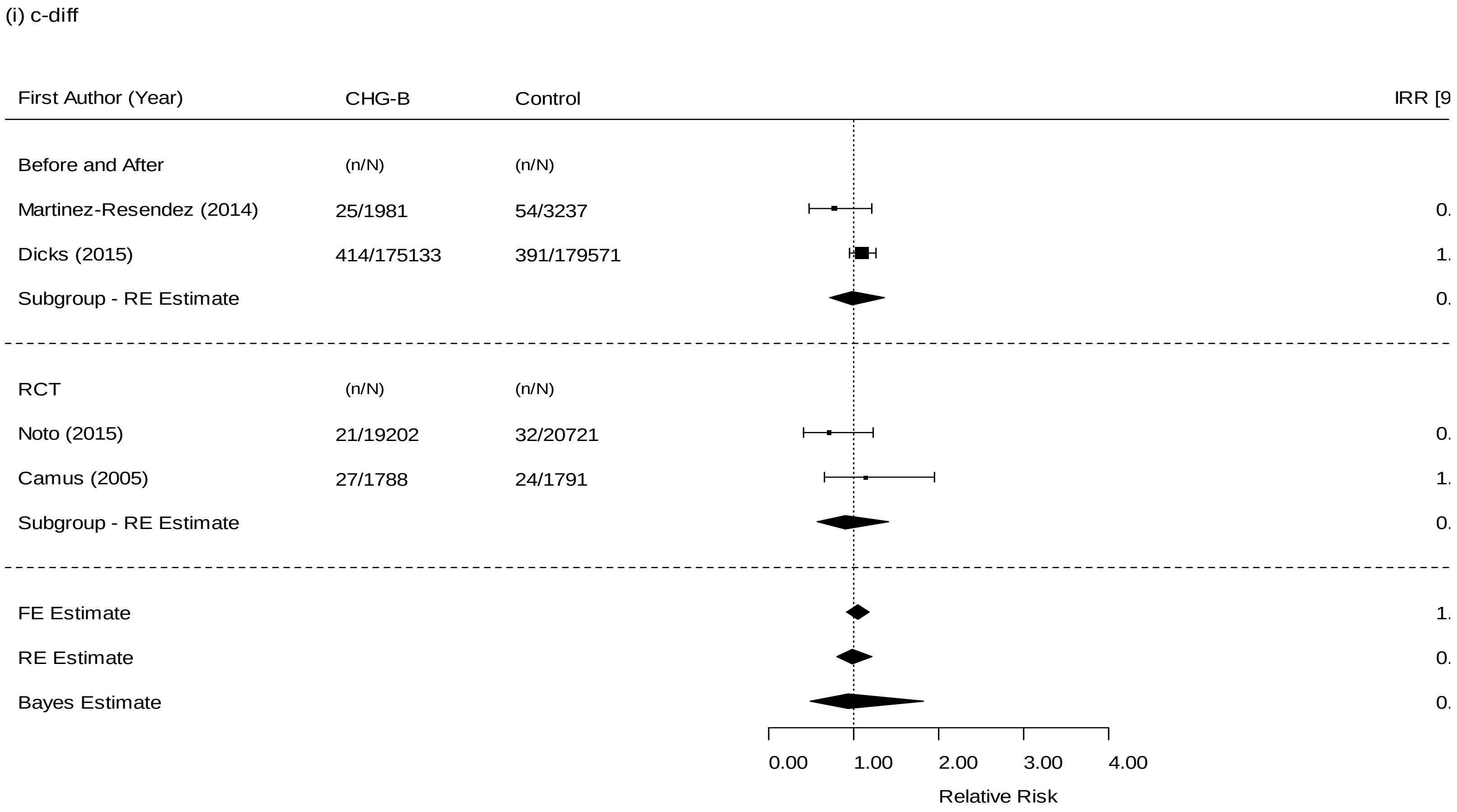

Figure S1. Forest plots presenting the effectiveness of CHG bathing in reducing: BSI (panel A), CLABSI (panel B), VAP (panel C), CAUTI (panel D), MRSA colonisation (panel E), MRSA associated BSI (panel F), VRE colonisation (panel G), VRE associated BSI (panel H), and Clostridium Difficile infections (panel I). FE = fixed effects, RE = random effects.
